# Editorial: Theme issue on the ecology of soil microorganisms

**DOI:** 10.1093/femsec/fiae032

**Published:** 2024-03-28

**Authors:** Petr Baldrian, Taina Pennanen, Petr Kohout, Hannu Fritze

**Affiliations:** Laboratory of Environmental Microbiology, Institute of Microbiology of the Czech Academy of Sciences, Prague 14200, Czech Republic; Natural Resources Institute Finland (Luke), Helsinki 00790, Finland; Laboratory of Environmental Microbiology, Institute of Microbiology of the Czech Academy of Sciences, Prague 14200, Czech Republic; Natural Resources Institute Finland (Luke), Helsinki 00790, Finland

## Abstract

Inoculation of common bean seed with diversified bacterial synthetic communities can induce deep modifications of both seed and seedling microbiota, even in living potting soil.

The fourth Ecology of Soil Microorganisms (ESM4) conference was held in Prague, Czech Republic in June 2022 as one of the very first microbiology meetings after the COVID pandemic. About 200 scientists gathered for a 5 day meeting in Prague. With 63 long and 43 short talks and one hundred posters the scientific community was kept busy from 9 am to 6 pm. The time of the COVID pandemic was difficult for science and probably everyone agrees that online meetings cannot replace the possibility to meet face to face. Although the post-pandemic time resulted in a lower number of participants (in the time of registration, it was not yet fully sure what exact hygiene measures would be needed), the enthusiasm of all participants that met after a long time of online only contacts was really high.

Since our first conference in 2011, we continue to see the increased appreciation of the role of microorganisms in ecosystem processes. In fact, it makes little sense to separate microbial ecology from the ecology of whole ecosystems as clearly demonstrated in the results from forest and agricultural contexts. Because microorganisms are essential agents and subjects of global change, particular attention was also paid to their possible roles under future climatic conditions.

As in the past, FEMS Microbiology Ecology offered its thematic issue as a peer reviewed publication venue for the results presented at the conference. We are happy that this resulted in 22 papers which give a good overview of the state of art and demonstrated the most pressing questions of current soil microbial ecology. The last years when high-throughput sequencing and other methodical developments became established helped to answer the major general questions about microbiome composition and demonstrated its essential role in ecosystem functioning. The present trend that can be seen in this thematic issue is to focus research on more practical questions, often those that evaluate alternative management strategies and try to assess their impact on ecosystems in general and their microbiomes in particular.

As usual, the agricultural soils, both croplands and grasslands, received the greatest attention. While the microbiome of the upper parts of the grassland soils is reasonably known, the deep layers were not frequently analyzed. Across a wide range of sites Guasconi et al. (2023) show that microbial biomass decreases with depth but diversity is mostly retained. Also the roles of saprotrophic fungi in grasslands is less known than in forests. Interestingly, Leifheit et al. (2024) indicate that the Ascomycota (and not the Basidiomycota, as in the forests) are the most efficient decomposers of litter-derived organic matter in this ecosystem. The effects of grazing is important not only for plants but also for soil microbiota, affecting both core microbiome and the more rare taxa (Tang et al. 2023). The herbivory not only affects the plant itself, but also its microbiome and volatilome (Lee Diaz et al. 2024). The effects of management were focused by three papers. While Peltoniemi et al. (2023) demonstrates the potentially positive effect of adding tree bark as a forestry by-product to soil to enhance its fungal communities and carbon content, Lori et al. (2023) show that organic farming including manure addition affects the microbiome. Manure addition, however, needs to be considered with caution since it can increase the spread of antimicrobial resistance genes (Sardar et al. 2023). What the future can be of wet grassland soils under global change is demonstrated by Edwards et al. (2023): higher plant productivity under future conditions with warmer climate and higher nutrient load may lead up to the switch of these ecosystems from C accumulators to C sources. Grasslands that are already affected should be restored—including their microbiomes. However, such transition may take more time than hoped (Barber et al. 2023).

Forests are another ecosystem that has recently received particular attention due to their ability to capture and store C but also due to their vulnerability to climate change effects (Baldrian et al. 2023). Recycling of nutrients in forests is driven by microorganisms and the rate of this process and the participating microbes depend on litter type, likely reflecting chemical composition (Min et al. 2023). Current forest management largely relies, to minimize costs of operation, on total removal of trees—clearcutting. Its deleterious effect on symbiotic soil fungi was demonstrated. In their paper, Martinović et al. (2022) show that bacteria are much less affected by tree removal and may thus help to maintain ecosystem functioning on clearcuts. On the other hand, drought effects, especially prolonged droughts over multiple years affect bacterial communities more profoundly than fungal communities (Jaeger et al. 2023). Last decade sees an increasing frequency of forest disturbances including insect infestations. Choma *et al*. (2023) show how surviving trees help to maintain the diversity of ectomycorrhizal root-symbiotic fungi and thus contribute to stand regeneration. Deadwood retention is one of the measures to maintain forest ecosystem diversity. In their paper, Bosch et al. (2023) show that this is due to the presence of several taxa of fungi and bacteria even within small volumes of deadwood. In addition to deadwood, also tree phyllosphere exhibits high beta-diversity due to the strong host tree selection of leaf-associated microorganisms (Yang *et al*. 2023) and is worth further investigation.

From the perspective of global change, peatlands are perhaps even more important than forests. They store more carbon but may be also important sources of greenhouse gasses (GHG). Their microbiology is, unfortunately, only partly understood. Weil et al. (2023) show that bacterial communities are vertically stratified even considering members of a single archaeal order. While there are concerns that GHG fluxes may increase from peatlands, this may not necessarily be the case since their production can be limited by the availability of terminal electron acceptors (Song et al. 2023). Also alpine treeline ecosystems belong to those under high impact of global change. Unfortunately, the ecosystem shift connected to tree colonization of higher altitudes may lead to C loss (Moravcová et al. 2023).

Interactions of microorganisms are often complex and range from symbiosis to antagonism. In the ectomycorrhizal system of eucalyptus with its ectomycorrhizal fungal partner *Pisolithus microcarpus*, C flow to fungal biomass seems to mostly reflect the demand of the fungal partner (Stuart et al. 2023). In another symbiotic system, Medina et al. (2023) show that social amoeba can rescue its bacterial host from interspecific competition. Viral shunts are events where bacteria are lysed after induction of the lytic cycle of the virus. Heffner et al. (2023) show that antibiotic addition can trigger this event and thus affect bacterial communities through activating their viruses.

The paper of Kopecky et al. (2023) tries to extend our general understanding of environmental factors from microbiome composition to its function. They show that soil properties—pH and organic matter content—not only affect microbiome composition, but also its function—metabolic profiles of its individual members, soil actinomycetes in this particular case.

Overall, this thematic issue shows that microbial ecology has the potential to increase our understanding of future of microbiomes and help us to manage ecosystems in a more sustainable manner. Considering the rapid changes of environmental conditions, these topics will remain highly important in the coming years. We are already looking forward to the developments that will be presented at the 5th Ecology of Soil Microorganisms conferences that will be held in Helsinki in 2025.
